# *Agrimonia pilosa*: A Phytochemical and Pharmacological Review

**DOI:** 10.1155/2022/3742208

**Published:** 2022-04-29

**Authors:** Tianyu Jin, Li Chi, Chongyang Ma

**Affiliations:** ^1^Class 1, Grade 2018 “5+3” Year Program Clinical Medicine, School of Basic Medicine, Capital Medical University, Youanmenwai, Xitoutiao, Fengtai District, Beijing 100069, China; ^2^School of Traditional Chinese Medicine, Capital Medical University, Youanmenwai, Xitoutiao, Fengtai District, Beijing 100069, China

## Abstract

*Agrimonia pilosa* Ledeb., which belongs to Agrimonia and Rosaceae, is used in traditional Chinese medicine. It exhibits excellent medicinal properties and has been used to treat various diseases, such as tumors, trichomoniasis, vaginitis, diarrhea, and dysentery. Phytochemical studies have revealed that *Agrimonia* has over 100 secondary metabolites that can be categorized into six classes, i.e., flavonoids, isocoumarins, triterpenes, phloroglucinol derivatives, tannins, and organic acids. This review summarizes recently published literature on the chemical structures of 90 bioactive compounds that have been identified in *A. pilosa* and examines their pharmacological properties, including their antitumor, anti-inflammatory, antioxidant, antibacterial, and antidiabetic properties, as well as the potential development of parasitic resistance to these chemicals. This review highlights existing knowledge gap and serves as a basis for developing novel preparations of *A. pilosa* with medicinal value.

## 1. Introduction

*Agrimonia pilosa* Ledeb, also known as agrimony, Agrimoniae herba, or hairyvein agrimony, belongs to Rosaceae. It is a perennial herb that grows in east Asia, central Europe, and the former Soviet Union. It is 50–100 cm long and covered with white pilose; it has a cylindrical (diameter of 4–6 mm) red brown lower part and a square columnar, slightly concave on all sides, and also has a green brown upper part with longitudinal grooves, ridge lines, and knots. It has a light, hard, easy-to-break, and hollow stem. It also has odd pinnate compound leaves that show alternate, dark green, wrinkled, and curled structures. Its leaves are brittle and fragile and have two sizes, alternating on the leaf axis. Top leaflets are large, and complete leaflets are oval or long oval after flattening. The apex is sharp, the base has a wedge shape, and the edge is serrated. Leaves have two amplexicaul and obliquely ovate stipules. The raceme is slender, the lower part of the calyx is tubular, the upper part of the calyx tube has barbs, the apex is five lobed, and petals are yellow. The fruit is 0.7–0.8 cm long and 0.3–0.4 cm in diameter and tastes slightly bitter.

In traditional Chinese medicine (TCM), *A. pilosa* is considered to have hemostasis-regulating, antimalarial, antidiarrheal, detoxification, and complement deficiency-restoring effects. It is often used to treat tumors, vaginal trichomoniasis, diarrhea, and dysentery. “ZhongHuaBenCao” (Chinese Materia Medica) recorded that the compatibility of *A. pilosa* with Arborvitae leaves and lotus root can treat hemoptysis and hematemesis. Rhizoma Imperatae and Jiaoshanzhi are used for gingival bleeding, and Daji, Mutong, and Rhizoma Rhizoma are administered for hematuria. “National Compendium of Chinese Herbal Medicine” documents that the double concentrate of the whole plant is externally used to treat *Trichomonas* vaginalis infection. Phytochemical studies have revealed that flavonoids, isocoumarins, triterpenes, tannins, organic acids, and phloroglucinol derivatives constitute the main classes of active ingredients in *A. pilosa* and might be responsible for its antioxidant, antifatigue, antitumor, hypoglycemic, cardioprotective, and hepatocyte protection effects [1–5].

Several reviews have been published over the past 20 years.  Table 1 summarizes all the reviews related to A. pilosa found in the scientific literature.

We noted that the existing reviews related to *A. pilosa* in major databases are mostly summaries of a single component or relatively brief short reviews, but an analysis of pharmacological activities of specific monomers and a discussion of related mechanisms are lacking. In this paper, to further understand the current research status of *A. pilosa* and provide justification for in-depth investigation and comprehensive application, we review the phytochemistry and pharmacological activities of *A. pilosa* and its possible mechanisms of action.

## 2. Methodology

The literature was reviewed by consulting scientific databases, including Web of Science, Springer, PubMed, ScienceDirect, and China National Knowledge Infrastructure. Plant taxonomy was confirmed via The Plant List. The query was supplemented by searching the reference lists of papers included in the first selection. The search terms were as follows: “Agrimonia pilosa” alone or in combination with “chemistry,” “pharmacology,” and “toxicity.” For this review, articles written in English or Chinese were taken into consideration.

## 3. Bioactive Compounds

Series bioactive compounds, including triterpenes and their glycosides, phloroglucinol derivatives, flavonoids and their glycosides, tannins, organic acids, and isocoumarins, have been isolated from *A. pilosa*. The two main classes include triterpenes and their glycosides and phloroglucinol derivatives. In this part, we summarized information about the main natural products isolated from *A. pilosa* over the past decade.

### 3.1. Flavonoids

Flavonoids identified in the *A. pilosa* extract mainly include quercetin, isoquercitrin, quercitrin, rutin, hyperoside, pilosanol A–C, and agriflavone (see Table 2 for details); these flavonoids exhibit significant antitumor, hepatocyte protection, free radical-scavenging, antioxidant, and immunoregulatory effects.

### 3.2. Isocoumarins

Isocoumarins identified in *A. pilosa* extracts mainly include agrimonolide, agrimonolide-6-O-*β*-D-glucopyranoside, and desmethylagrimonolide (Table 3). They exhibit hepatocyte protection, anti-inflammatory, and antitumor activities; they also regulate blood glucose and reduce insulin resistance (IR) [35–37].

### 3.3. Triterpenes

Triterpenes are the main chemical constituents of *A. pilosa*. Several bioactive monomers, such as corosolic acid, euscaphic acid, ursolic acid, and pomolic acid, are also found in *A. pilosa* (Table 4). Most of *A. pilosa* triterpenes promote insulin sensitivity, improve glucose metabolism, and reduce oxidative stress in vitro, indicating that they have potential for the development of antidiabetic drugs [41].

### 3.4. Phloroglucinol Derivatives

Several phloroglucinol derivatives have been extracted from *A. pilosa*. For example, agrimol A, B, C, D, and E were isolated from *A. pilosa* petroleum ether extract by the Shanghai Institute of Materia Medica and the Shanghai Fourteenth Pharmaceutical Factory (1975) [42]. The phenolic compounds agrimol F and G were isolated from *A. pilosa* ethyl ether extract by Yamaki et al. [43]. Agrimophol and pseudoaspidin were isolated from the petroleum ether extract of *A. pilosa* rhizomes by Pei et al. [44]. Agripinol A–C were isolated from *A. pilosa* and named by Tang et al. [45] (Table 5).

### 3.5. Tannins and Organic Acids

Tannins and organic acids in *A. pilosa* mainly include potentillin, pedunculagin, casuarinin, isovanillic acid, and protocatechuic acid (Table 6), which exhibit antitumor, anti-inflammatory, and free radical-scavenging activities [51–53]. However, studies on the pharmacological activities of agrimony tannins have mostly focused on their components rather than specific monomers.

## 4. Pharmacological Activity

For about 100 hundred years, *A. pilosa* has been used in China for treating cancers, bleeding, diarrhea, and parasitic infections [54]. In this section, we summarize the pharmacological activities of *A. pilosa* (see Table 7, Table 8, Table 9, Table 10, Table 11, and Table 12 for details).

### 4.1. Antitumor Activity

Total flavonoids derived from *A. pilosa* exhibit antitumor effects in a concentration-dependent manner against several tumor cell lines, including MKN-45 human gastric cancer cells, HepG2 human hepatoma cells, U266 human multiple myeloma cells, MCF-7 human breast cancer cells, A549 human non-small cell lung cancer cells, and HeLa cells, with an IC50 of 127.50, 53.31, 202.10, 206.80, 54.17, and 170.40 *μ*g/mL, respectively [63, 74]. Catechin, luteolin, quercetin, quercitrin, hyperoside, rutin, and luteolin 7-O-*β*-glucoside exhibit significant 2,2-diphenyl-1-picrylhydrazyl (DPPH) free radical-scavenging activity, with an IC50 of 5.06, 7.29, 4.36, 7.12, 6.34, 6.36, and 8.12 *μ*M, respectively [3].

Gastric cancer is one of the most common malignant tumors. Studies have shown that quercetin, a flavonoid isolated from *A. pilosa*, can sensitize human gastric adenocarcinoma cell lines (AGS) to SN-38, a DNA topoisomerase I inhibitor; the cell viability and apoptosis rates induced by the combination of quercetin and low-dose SN-38 are similar to those obtained with a high-dose SN-38 alone. In vivo, the combined activity of quercetin and SN-38 induces the downregulation of the concentrations of the vascular endothelial growth factor (VEGF)-A and the VEGF-receptor 2; it also decreases the percentage of Tie2-expressing monocytes in AGS xenograft mice compared to control mice [55]. In addition, agrimonolide exhibits a dose-dependent apoptosis-inducing effect in AGS cells with an IC50 of 25.9 *μ*mol/L; its underlying mechanism involves the B-cell lymphoma-2 (Bcl-2)/Bcl-2-associated X (Bax) and mitogen-activated protein kinase (MAPK) pathways and occurs through the regulation of Box Bcl-2/Bax and protein kinase 1/2 expression, p38 phosphorylation, and caspase-3 protease activation, thereby promoting apoptosis in AGS cells [56]. At 100 *μ*mol/L, beta-carotene induces apoptosis and DNA fragmentation in AGS cells by promoting p53 and Bcl-2 expression [1, 2].

Agrimol B, a phloroglucinol derivative isolated from *A. pilosa*, causes the arrest of prostate and A549 lung cancer cells at the G0 phase by decreasing cellular myelocytomatosis viral oncogene (c-MYC) and SKP2 expression, promoting p27 expression, and downregulating SPT16 and SSRP1 expression. Oral administration of agrimol B (10 mg/kg) inhibits tumor growth in mice injected with human prostate cancer cells, but it does not significantly modify their body weight [57]. Ellagic acid also arrests A549 cells at the G0 phase [57]. Prostate cancer is the second-most frequent cancer and the fifth leading cause of cancer-related deaths in men [75]. Agrimol B efficiently inhibits the proliferation of prostate cancer cells. It also decreases c-MYC and SKP2 expression and increases p27 expression in PC-3 cells, thereby inhibiting mitosis in these cells [57]. Moreover, ellagic acid arrests PC-3 cells at the G0 phase [57]. Apigenin induces apoptosis in PC-3 cells and significantly reduces tumor size by inhibiting class I histone deacetylases (HDACs), especially HDAC1 and HDAC3 [58, 76].

Among flavonoids, quercetin shows the strongest cytotoxicity to MCF-7 breast cancer cells [59]. Agrimophol elicits a concentration-dependent inhibitory effect against K562 human chronic myelogenous leukemia cells; particularly, 10 mg/mL agrimophol induces cytotoxicity similar to 50 IU/mL vincristine (*P* > 0.05) [60]. Agripinol A–C have more significant cytotoxic effects on HCT-116, MDA-MB-231, and PC-3 cells than 5-fluorouracil [45].

Total *A. pilosa* tannins exhibit antitumor activities against HeLa, MCF7, and SPC-A-1 human lung adenocarcinoma cells [77]. In vitro, tannin extracts induce apoptosis in Bel-7402 and HepG2 cells by regulating free Ca^2+^ level overloading and increasing reactive oxygen species levels [78]. In vivo, the total tannin in *A. pilosa* extract inhibits the growth of S180 sarcoma [78]. The methanolic extract of *A. pilosa* can inhibit the invasion and metastasis of HT1080 cells by inhibiting the expression activity of MMP-2 and MMP-9 through ERK, JNK, and AKT-1 inactivation [79]. Methanolic *A. pilosa* extract shows obvious cytotoxicity at 10 *μ*M, and by 34% at 20 *μ*M in IMR90 cells, higher than the cytotoxicity to HT1080 cells at the same concentration. Therefore, the pure alcohol extract is limited by poor selectivity and strong cytotoxicity. Active components should be further elaborated and subjected to in vivo experiments. Notably, 10 *μ*g/ml alcohol extract of *A. pilosa* shows obvious cytotoxicity (*P* < 0.001), and 20 *μ*g/mL alcohol extract of *A. pilosa* shows a cytotoxicity of 34% against IMR90 cells, higher than the cytotoxicity against HT1080 cells at the same concentration. Similar to pure alcohol extract, alcohol extracts at different concentrations are limited by poor selectivity and strong cytotoxicity; therefore, the active components of *A. pilosa* extracts should be further elucidated. Ellagic acid inhibits proliferation and metastasis and induces apoptosis in several tumor cells, thereby eliciting broad-spectrum antitumor effects [80]. Agrimoniin, a tannin found in *A. pilosa*, exhibits antitumor activity against ascite- and solid-type rodent tumors in mice, and the underlying mechanism may involve the enhancement of the host's immune response [61]. The combination of quercetin and hyperoside at a 1 : 1 ratio inhibits 786‐O renal cancer cell proliferation by upregulating the expression of zinc finger and BTB domain containing 10 (ZBTB10) and downregulating the mRNA expression of Sp1, Sp3, and Sp4 [62].

In summary, *A. pilosa* extracts elicit inhibitory effects against several tumor cell types, and this finding provides a theoretical basis for developing *A. pilosa*-based antitumor therapies. The combination of *A. pilosa* constituents and classical chemotherapeutic agents may be potential treatment strategies against tumors.

### 4.2. Free Radical Scavenging and Antioxidant Activities

Metabolic processes lead to the production of large amounts of active oxygen free radicals. Free radicals in humans have been linked to the deterioration of chronic diseases, such as diabetes, tumors, and Alzheimer's disease (AD).

The methanolic extract of *A. pilosa* leaves protect nonlipid oxidative damage from various model systems, including liposome oxidation, deoxyribose oxidation, protein oxidation, metal ion oxidation, and hydrogen peroxide oxidation models [81]. Tannin extracts of *A. pilosa* roots exhibit dose-dependent DPPH free radical-scavenging and liposome peroxidation inhibitory effects in vitro [15]. Total *A. pilosa* flavonoids elicit dose-dependent antioxidant effects, with a ferric reducing antioxidant potential assay value of 56.87 mmol/L FeSO_4_ [63]. In addition, *A. pilosa* flavonoids exhibit significant scavenging effects against DPPH, 2ʹ-azinobis-(3-ethylbenzthiazoline-6-sulphonate), and hydroxyl radicals [47, 64]. The free radical-scavenging activities of these flavonoid extracts may be attributed to the presence of quercetin and hypericin, and one of the possible mechanisms underlying this effect is the activation of the Sonic hedgehog signaling pathway [82]. Tannins from *A. pilosa* also scavenge DPPH free radicals in a dose-dependent manner and inhibit liposome peroxidation activity. Protocatechuic acid exhibits significant free radical-scavenging activity, especially against DPPH• and O2 [65]. A DNA nicking assay has revealed that taxifolin, catechin, hyperoside, quercitrin, and rutin protect against oxidative DNA damage. Based on the structure-function relationship provided by quantum chemistry theory, glycosylation at C-6 enhances the antioxidant activity of flavonoids by rendering a uniform distribution of spin density and improving free radical stability. These findings may serve as a theoretical basis for designing and developing antioxidant preparations [3]. Antioxidant activity is one of the main activities of *A. pilosa* tannins and organic acids. In D-galactose-induced subacute aging mice, *A. pilosa* tannin extract elicits an antioxidant activity by increasing superoxide dismutase activity and decreasing malondialdehyde (MDA) activity in blood [15]. This antioxidant activity may be due to protocatechuic acid, protocatechuic aldehyde, and gallic acid as the main monomers. Protocatechuic acid protects damaged rat liver cells through menadione by enhancing its antioxidant capacity and stage II enzyme activity through the Nrf-2 pathway [66]. In H9c2 cell exposed to hypoxia-induced oxidative stress, agrimonolide maintains mitochondrial homeostasis, thereby reducing oxidative stress damage to mitochondria. Moreover, agrimonolide promotes cell proliferation by regulating the cell cycle and inhibits H9c2 apoptosis by reducing caspase 3 and Bax and promoting Bcl 2. Autodock software predicts that Tom20 protein may be a potential target of agrimonolide, but the precise mechanism of agrimonolide and Tom20 interaction needs further research [83].

### 4.3. Anti-Inflammatory Activity

**Figure 1:**
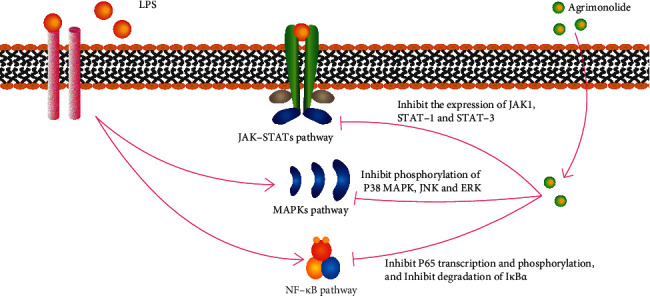
Anti-inflammatory mechanism of agrimonolide [35, 84, 85].

*A. pilosa* isocoumarins play a beneficial anti-inflammatory role by scavenging intracellular nitric oxide (NO), inhibiting cyclooxygenase-2 (COX-2) and inducible NO synthase (iNOS) transcription and translation, and reducing the expression of inflammatory cytokines, such as tumor necrosis factor-*α* (TNF-*α*) and interleukin (IL) 6. These activities are correlated with the presence of agrimonolide and agrimonolide-6-O-*β*-D-glucopyranoside, especially with that of agrimonolide [33, 35, 47, 55]. In lipopolysaccharide (LPS) induced RAW 264.7 macrophages, agrimonolide inhibits NO release in a dose-dependent manner by reducing IL-1, IL-6, and TNF-*α* levels and inhibiting iNOS activity [47]. The mechanisms underlying the anti-inflammatory activity of agrimonolide involve three signaling pathways. First, it inactivates nuclear factor-kappa B by inhibiting p65 transcription and phosphorylation and preventing LPS-induced I*κ*B*α* degradation; second, pretreatment with agrimonolide prevents LPS-induced P38 MAPK, C-Jun *n*-terminal kinase (JNK), and extracellular regulated protein kinase phosphorylation; and third, it reduces the LPS-induced production of phosphorylation proteins, such as Janus kinase 1, signal transducer and activator of transcription (STAT)-1, and STAT-3, thereby blocking inflammatory signaling cascades (Figure 1) [35, 84, 85].

The ethanol extract of agrimony can inhibit xylene-induced ear edema in mice and carrageenan-induced paw edema in rats, and tiliroside has been proved to be the main active ingredient. Moreover, *A. pilosa* tiliroside significantly inhibits the overproduction of NO, downregulates the LPS-induced overexpression of iNOS and COX-2, and inhibits the phosphorylation of JNK and p38 proteins in LPS-activated RAW 264.7 macrophages; these results suggest that the anti-inflammatory mechanism includes the downregulation of iNOS and COX-2 protein and the inactivation of mitogen-activated protein kinase (MAPK)/JNK, in addition to the MAPK/p38 signaling pathway [86].

Phloroglucinol derivatives also exhibit anti-inflammatory activities [67]. In RAW 264.7 macrophages, pilosanol N inhibits NO production by inhibiting the expression of iNOS and the expression of COX-2; it also induces the elimination of NO and nitrogen free radicals generated by the NO donor 4-ethyl-2-hydroxyamino-5-nitro-3-hexenamide.

The mixed extract from *A. pilosa* and *Salvia miltiorrhiza* Bunge alleviates gouty arthritis [87]. In terms of analgesic effects, one off administration and one-week treatment reduce the pain threshold in a dose-dependent manner (from 10 mg/kg to 100 mg/kg) in a mono-iodoacetate (MIA) induced osteoarthritis (OA) model. In terms of anti-inflammatory activity, the mixed extract reduces plasma TNF-*α*, IL-6, and CRP levels in MIA-induced osteoarthritis and ameliorates the progress of 2.5% croton oil-induced ear edema in mice. In LPS-stimulated RAW 264.7 cells, the mixed extract inhibits the release of NO, PGE2, LTB4, and IL-6 and increases PPAR*γ* phosphorylation of proteins in a concentration-dependent manner (from 1 *μ*g/mL to 100 *μ*g/mL). In most experiments, the effects induced by the mixed extract are almost equal to or higher than those induced by *Perna canaliculus* powder. However, the limitation of this study is that the major active ingredient in the mixed extract is unknown, and the potential mechanism of its analgesic effect should be further analyzed.

### 4.4. Antidiabetic Activity

T2DM is characterized by insulin and leptin resistance. Insulin levels are regulated by protein tyrosine phosphatase (PTP) 1B, a key member of the PTP family, which decreases insulin sensitivity [88]. PTP1B is considered an important target for the treatment of T2DM and obesity. Nguyen et al. [29] isolated agriflavone and kaempferol-3-O-((S)-3-hydroxy-3-methylglutaryl(1 ⟶ 6))-*β*-d-glucoside, which are two new flavonoid glycosides, and 16 known compounds—kaempferol 7-O-*β*-D-glucoside, kaempferol 7-O-*β*-D-glucuronide, kaempferol 3-O-*β*-D-glucoside, apigenin, apigenin 7-O-*β*-D-glucoside, apigenin 7-O-*β*-D-glucuronide, quercetin, quercetin 3-O-*β*-D-glucoside, quercetin 3ʹ-O-*β*-D-glucoside, luteolin 7-O-*β*-D-glucoside, luteolin 7-O-*β*-D-glucuronide, luteolin 7-O-*β*-D-glucuronide methyl ester, luteolin 7-O-*β*-D glucuronide butyl ester, luteolin 3ʹ-O-*β*-D-glucoside, ellagic acid, and dihydrodehydro-diconiferyl alcohol 9ʹ-O-3-D-glucoside—from the aerial parts of *A. pilosa* and evaluated their inhibitory effects on PTP1B. They found that apigenin 7-O-*β*-D-glucuronide and ellagic acid inhibit the PTP1B activity with IC50 of 7.14 ± 1.75 and 7.73 ± 0.24 *μ*M, respectively [29]. However, PTP1B inhibitors are limited by their high anionic charge that prevents their binding affinities; the bioavailability of apigenin 7-O-*β*-D-glucuronide, which has a carboxylic acid group, can be improved by using its methyl or ethyl derivative; therefore, it is a potential natural T2DM inhibitor.

Postprandial hyperglycemia is closely related to T2DM progression [64, 89, 90]. In a clinical study, *A. pilosa* powder reduces the incidence T2DM-related complications by reducing high postprandial hyperglycemia, showing its potential for use in T2DM treatment [36]. Flavonoids and isocoumarins present in *A. pilosa* may be responsible for such postprandial hyperglycemia-reducing effects.

*α*-Glycosidase is involved in glycogen decomposition and glucose regulation. Total *A. pilosa* flavonoids inhibit *α*-glucosidase activity, and the active components are luteolin, quercetin, vitexin, and isovitexin; among them, quercetin has the highest activity and noncompetitively inhibits *α*-glucosidase [30, 64]. In addition, four isocoumarins, agrimonoide, agrimonolide-6-O-*β*-D- glucopyranoside, desmethylagrimonolide, and desmethylagrimonolide-6-O-*β*-D-glucopyranoside, were found to be *α*-glucosidase inhibitors. Moreover, the endogenous glucose-inhibitory activity of agrimonolide is related to the inhibition of phosphoenolpyruvate carboxykinase, which is the rate-limiting enzyme in the gluconeogenesis pathway (IC50, 8.3 *μ*mol/L) [68]. In insulin-resistant cells, agrimonolide improves insulin sensitivity and promotes insulin-mediated glycogen synthesis. Agrimonolide significantly improves glucose uptake in IR cells and exhibits the highest hypoglycemic activity; glucose consumption in HepG2 cells is 62.3% lower than that of 3 mM at a concentration of 20 *μ*M agrimonolide, which is not significantly different from that obtained with metformin (70.5%) [37].

### 4.5. Lipid Metabolism Regulation Activity

Obesity is significantly associated with the pathogenesis of IR, metabolic syndrome, and T2DM. Lipid metabolism and adipose tissue inflammation are partly responsible for the development of obesity-induced IR [91]. Thiazolidinediones, which are *A. pilosa* triterpenoids, have better insulin sensitization effects and lower lipid formation effects on 3T3-L1 cells than classical hypoglycemic drugs; they elicit these effects by regulating adiponectin and GLUT4 mRNA expression through the upregulation of upstream genes, such as peroxisome proliferator-activated receptor *γ* (PPAR*γ*), SREBP-1, and C/EBP*α*. These findings indicate that *A. pilosa* triterpenoids may be potential natural drugs for the treatment of IR and T2DM [69] and improve fat metabolism.

In high-sugar-concentration-induced IR-HepG2 cells, *A. pilosa* triterpenes improve glucose metabolism [41], decrease reactive oxygen species levels, promote superoxide dismutase release, reduce malondialdehyde content, and activate the nuclear factor-E2-related factor 2 (Nrf2) antioxidative response element signaling pathway, thereby ameliorating oxidative stress in these cells. Moreover, they reduce JNK expression and phosphorylation and promote insulin receptor substrate-1 (IRS-1) Ser 307 expression in these cells. Therefore, *A. pilosa* triterpenes ameliorate hyperglycemia in IR cells by improving oxidative stress and regulating the JNK and IRS pathways [41].

Silent mating-type information regulation 2 homolog 1 (SIRT1) is a key regulator of obesity-related metabolic pathways, and its deletion leads to obesity, metabolic dysregulation, and IR [92, 93]. Agrimol B inhibits adipogenesis in 3T3-L1 adipocytes at the early differentiation stage, with an IC50 of 3.35 *μ*M, and this effect is partly due to the stimulation of SIRT1 expression and the induction of the cytoplasm-to-nucleus SIRT1 shuttle. Agrimol B also inhibits 3T3-L1 adipocyte differentiation by inhibiting PPAR*γ*, C/EBP*α*, FAS, UCP-1, and apoE expression [70, 91].

*A. pilosa* aqueous extract (5.000 g/kg diet) can improve the glucose and lipid metabolism of ovariectomized rats under a high-fat diet and the degree of hepatic steatosis in rats. The aqueous extract of *A. pilosa* has no effect on the increase in body weight and food intake of rats, but it improves the postprandial high-glucose state of ovariectomized rats, reduces the level of serum total cholesterol and low-density lipoprotein, and improves the weight of liver and the degree of fatty liver. *A. pilosa* extract also improves insulin resistance and glucose metabolism by increasing adiponectin levels without affecting the expression of adiponectin receptor. Moreover, it increases serum insulin levels, but the molecular mechanism of promoting insulin secretion is unclear. It also inhibits the synthesis of fatty acids and cholesterol, inhibits the increase of liver quality induced by high-fat diet, and improves the degree of fatty liver by downregulating fat formation-related genes, such as fatty acid synthase, acetyl-coenzyme A carboxylase alpha, and 3-hydroxy-3-methylglutaryl-coenzyme; furthermore, *A. pilosa* extract inhibits the synthesis of fatty acids and cholesterol. In addition, the improvement of fatty liver is related to the increase in adiponectin levels and the improvement of insulin resistance [94]. However, a limitation of the present study is that estrogen-like activities of *A. pilosa* in postmenopausal metabolic syndrome models are not analyzed. In addition, the specific concentration and effective components of *A. pilosa* aqueous extract are not described. Therefore, studies should aim to isolate and characterize the functionality of each compound derived from *A. pilosa*.

### 4.6. Anthelmintic Activity

*A. pilosa* shows an anthelmintic activity. Agrimophol inhibits glycogen decomposition in tapeworm by directly coming in contact with the tapeworm's body, thereby inhibiting tapeworm aerobic and anaerobic metabolism [71]. Agrimol G destroys the parasite cuticle when it is incubated with adult *Haemonchus* parasites for 3 h. Microtubule degeneration and the presence of electron-dense and electron-lucent bodies around microtubules are not observed in *A. pilosa* and albendazole or ivermectin cotreatment; therefore, agrimol G elicits a killing effect on *Haemonchus* parasites by inhibiting microtubule aggregation [72]. Agrimonia essential oil (at concentrations of 10, 50, and 100 *μ*g/mL) shows a dose-dependent inhibitory activity on *Leishmania* promastigote and intracellular amastigote forms in vitro. Agrimonia essential oil at different concentrations have no toxic effects on host cells. The active chemical components of essential oil should be analyzed and purified [95].

Most antiparasitic drugs, such as chloroquine and albendazole, cause evident side effects, including fetal malformation. *A. pilosa* extracts, as natural products, have a strong antiparasitic effect with relatively low toxicity. Therefore, the effects of *A. pilosa* extracts on embryonic development and pharmacokinetics should be studied to provide a basis for developing safe antiparasitic drugs for pregnant women. In addition, the in vivo immune stress mechanism of *A. pilosa* to prevent and treat parasitic infection should be evaluated.

### 4.7. Others

#### 4.7.1. Anti-Alzheimer's Disease Activity

The mechanism underlying AD development is closely associated with amyloid-*β* aggregation and neurotic plaque formation, which causes neurotoxicity and accumulation of neurotic plaques in the brain. In *β*-amyloid-infused rats, the administration of 2% *A. pilosa* lyophilized aqueous extracts in a high-fat diet (43% energy as fat) induces a reduction in neuro-inflammation, prevents hippocampal amyloid-*β* accumulation, and enhances hippocampal insulin signaling, thereby effectively preventing cognitive dysfunction and improving hippocampal IR [96]. The loss of brain cholinergic function causes memory impairment in patients with AD, and AchE is involved in the termination of the cholinergic signal by playing an important role in acetylcholine hydrolysis. Sixteen flavonoids extracted from the aerial parts of *A. pilosa* exhibit moderate inhibitory effects against AchE in vitro, suggesting that flavonoids from *A. pilosa* may be natural agents for AD treatment [29]. Among the 10 flavonoid glycosides (1–10) isolated from the part of crane grassland [31], compounds 1 and 4 have no activity, and the other compounds show a moderate acetylcholinesterase inhibitory activity. IC50 ranges from 76.59 ± 1.16 *μ*M to 97.53 ± 1.64 *μ*M, which supports the above conclusion.

#### 4.7.2. Hepatocyte Protection Activity

The aqueous extract of *A. pilosa* improves the development of fatty liver in a high-fat diet model [90, 94]. A high-fat diet increases the expression of inflammatory cytokines in the adipose tissue and liver of rats, whereas the aqueous extract of *A. pilosa* (0.1%) supplement inhibits the increase of liver weight and improves the degree of the fatty liver of rats. *A. pilosa* aqueous extract also improves the impaired glucose tolerance of rats caused by high-fat diet and reduces the blood glucose level of rats, suggesting that *A. pilosa* aqueous extract can improve insulin resistance. The specific mechanism is related to the inhibition of liver and adipose tissue inflammation and the improvement of insulin resistance by reducing the expression of the rat liver inflammation-related genes G6PD and IL1B and the levels of the serum inflammatory cytokines IL-6 and TNF-*α* [90].

Isocoumarinic compounds may be responsible for the hepatocyte protection activity of *A. pilosa* as they improve oxidative stress. Agrimonolide, the main active isocoumarin in *A. pilosa*, protects rat primary hepatocytes by inhibiting oxidative stress induced by tacrine and tert-butyl hydrogen peroxide [5]. Agrimonolide and demethylated agrimonolide reduces oxidative stress in HepG2 cells by inducing heme oxygenase-1 and Nrf2 expression and inhibiting Kelch-like ECH-associated protein 1 expression [73]. Recently, five new dimeric phloroglucinol derivatives, namely, agrimones A−E, have been isolated from the whole plant of *A. pilosa*. Among them, 10 *μ*M agrimones A, D, and E show a moderate liver protective activity in p-n-acetyl-p-aminophenol-induced HepG2 cell and increase the cell viability from 62.09% to 70.66%, 67.21%, and 69.21%, respectively [48].

A. pilosa ethanol extract exerts the protective effect on LPS-induced cell damage in human HepG2 hepatocytes through antioxidant and anti-inflammatory activities. The mechanism involves *A. pilosa* extract (100 and 200 *μ*g/mL) that dose dependently reduces the production of intracellular reactive oxygen species stimulated by LPS to the basal level, reverses the expression of glutathione peroxidase gene and protein inhibited by LPS, and has no cytotoxic effect at the experimental dose. However, only in vitro experiments have been performed; although the content and proportion of various components in the extract are determined, the components mainly related to the above hepatocyte protective activity remain unknown [42].

#### 4.7.3. Antimicrobial Activity

Several phloroglucinol derivatives present in *A. pilosa* have antibacterial activities. For example, agrimol C, agrimol F, agrimol G, and agrimophol completely inhibit the growth of methicillin-resistant *Staphylococcus aureus*, *Bacillus cereus*, and *Gardnerella* species [46, 97]. However, experiments on the antibacterial activity of phenolic components of *A. pilosa* were performed in 1988, and the specific mechanism has not been clarified. Considering the possibility of bacterial variation and drug resistance, the antibacterial activity of phenolic compounds in *A. pilosa* should be further investigated (see Table 13).

#### 4.7.4. Antiviral Activity

The mixture of *A. pilosa* and gallnut extract (APRG64) at a 6 : 4 ratio significantly inhibits the expression of HCV core 1b and NS5A proteins at a concentration of 5 *μ*g/mL in vitro. Further experiments have shown that 14 compounds isolated from the mixture inhibit the expression of these two proteins; the experimental concentration of 5 *μ*g/mL has no obvious cytotoxicity, but the inhibitory activity of luteolin is the most significant (*P* < 0.01) [98]. However, only in vitro experiments have been conducted, and experimental results have shown that the antiviral activity of a single *A. pilosa* extract is significantly weaker than that of gallnut and PRG64. Considering that all 14 compounds isolated from APRG64 can also be obtained from Agrimonia, this situation may be related to the relatively low content of anti-HCV active components in Agrimonia extract. The same group of researchers also studied the inhibitory effects of APRG64 on SARS-CoV-2 [99]. They found that ARGP64 strongly inhibits SARS-CoV-2 by interfering virus entry and replication. Further studies have revealed that the active components in the mixture are ursolic acid, quercetin, and 1,2,3,4,6-penta-o-gallol-*β*-D-glucose). These compounds (purity > 97%) show strong antiviral activities (reduction rate of 21.05% at 25 *μ*g/mL) against SARS-CoV-2; in particular, 1,2,3,4,6-penta-o-gallol-*β*-D-glucose entirely suppresses the formation of plaques at 1 *μ*g/mL and exhibits a potent antiviral activity at lower concentrations (0.125, 0.25, and 0.5 *μ*g/mL). Molecular docking analysis has shown that these components bind potently to the spike receptor-binding-domain (RBD) of SARS-CoV-2 and its variant B.1.1.7. These findings indicate *A. pilosa* and APRG64 as potent drug candidates for treating SARS-CoV-2 and its variants.

### 4.8. Estrogen-Like Effect

The aqueous extract of *A. pilosa* shows an estrogen-like activity in vitro. Its specific performance is described as follows. In a competitive binding experiment, apigenin hexose, luteolin glucosidic acid, and apigenin glucosidic acid in the aqueous extract of *A. pilosa* can bind to estrogen receptors (ERs) and display E2-bound ER*α* and ER*β*. In an E-SCREEN assay using MCF-7 cells, *A. pilosa* significantly stimulates MCF-7 cell proliferation at concentrations of 1 and 10 *μ*G/mL (*P* < 0.001) and does not show an antagonistic activity against E2 in MCF-7 cells when they are co-treated with E2. A. pilosa-stimulated proliferation is blocked by the addition of the estrogen antagonist ICI 182780. *A. pilosa* increases the mRNA expression of the estrogen response genes PS 2 and PR (*P* < 0.05) [100]. However, no animal experiments have been performed, and a single cell model was used. Another study [94] has presented a supporting conclusion via experiments on ovariectomized rats cultured on a high-fat diet although more direct and persuasive experiments, such as effects of *A. pilosa* on estrogen receptors and the uterus in postmenopausal syndrome models, have not been performed.

### 4.9. Analgesic Activity

The analgesic properties of *A. pilosa* extract have been examined in ICR mice. In tail flick (*P* < 0.05), hot plate tests and esthetic acid-induced writing test, 200 mg/kg *A. pilosa* extract elicits different degrees of pain relief. As for the test on nociceptive behavior induced by substance P (0.7 *μ*g/5 *μ*L), 200 mg/kg *A. pilosa* extract administered orally for 30 min prior to the substance P intrathecal injection significantly reduces the cumulative nociceptive response time of mice. *A. pilosa* also elicits an analgesic effect on yohimbine (*α*2-adrenergic receptor antagonist) that decreases during intraperitoneal pretreatment. It is not affected by naloxone (opioid receptor antagonist) or mexiletine (5-HT serotonergic receptor antagonist), suggesting that this analgesic effect may be mediated by *α*2-adrenergic receptor but not by an opioid receptor or serotonergic receptor [101]. However, the specific extraction method of *A. pilosa* extract has not been described, and the concentration has not been specified. Therefore, only qualitative experiments can be performed, and the analgesic effect intensity of Agrimonia extract cannot be determined.

The tannin component (0.375 g/kg) in the water extract of *A. pilosa* regulates rhythm in a desynchronosis model. This chronic effect is determined by the lithium dose, corresponding lithium concentration in the brain, and nature of lithium carriers; a dose-dependent effect is clearly observed, but lithium-depleted acute extract with a high dose (10 times) is not absorbed in the rat intestine and does not show rhythm mediation, suggesting that lithium ions in *A. pilosa* tannin are more easily absorbed by the intestine and can pass through the blood-brain barrier to regulate rhythm. However, the pharmacokinetic mechanism of lithium ion in *A. pilosa* tannin remains to be analyzed. Further research may provide ideas for the development of wide treatment windows and highly selective psychotropic drugs [102].

## 5. Conclusions and Prospects

In China, *A. pilosa* has been applied to treat diseases for hundreds of years. Although systematic toxicology research has not been performed, no obvious toxic reactions caused by *A. pilosa* have been reported in the cases of clinical application of *A. pilosa* formula. In all reported animal experiments, oral administration or injection of agrimony extract does not cause weight loss in experimental animals compared with that in control animals.

Numerous in vitro or animal experiments on the pharmacological activities of *A. pilosa* have been conducted, but the clinical application of *A. pilosa* preparation is mostly described in simple case reports; and systematic case-control studies, clinical control experiments, or cohort studies have not been conducted. Among pharmacological activities that have been reported, the strong sensitizing effect of quercetin on irinotecan should be investigated. The combination of quercetin and irinotecan may become one of the effective means to reduce the serious adverse reactions caused by large irinotecan doses. *A. pilosa* extract has great potential for regulating lipid metabolism and treating T2DM. As an edible medicinal plant, *A. pilosa* can be conveniently applied with oral hypoglycemic agents to treat patients with obesity and T2DM. The high safety of *A. pilosa* has also ensured its application in the treatment of T2DM without any additional adverse reactions. The effects of *A. pilosa* on fetal teratogenesis and development should also be analyzed to provide a basis for developing antiparasitic drugs for pregnant women. Considering the emergence of multidrug-resistant bacteria due to the widespread use of antibiotics, experiments on antibacterial active components from *A. pilosa* against common drug-resistant bacteria are also needed.

Although *A. pilosa* has been extensively studied, further research should be conducted to clarify the accurate correlation between phytochemical and pharmacological profiles and evaluate the pharmacokinetic and pharmacodynamic interactions of active components. This review summarized the available information on *A. pilosa* and provided evidence of activity; therefore, it may contribute to the development of new medicinal formulations.

## Figures and Tables

**Table 1:** Previous reviews.

Year of publication	Main topic	Years surveyed	Limitations	References
2003	Pharmacology and ethnomedicine	NS	Short review	[6]
2004	Pharmacology and ethnomedicine	NS	Short review	[7]
2006	Phytochemistry and pharmacology	NS	Short review	[8]
2008	Phytochemistry and pharmacology	NS		[9]
2008	Phytochemistry and pharmacology	NS	Short review	[10]
2009	Phytochemistry and pharmacology	NS	Only the antitumor activity was discussed	[11]
2011	Botany, phytochemistry and pharmacology	NS	Only the antitumor activity was discussed	[12]
2011	Phytochemistry and pharmacology	NS	Only the antioxidant activity was discussed	[13]
2015	Phytochemistry and pharmacology	NS	Short review	[14]
2016	Phytochemistry and pharmacology	NS		[15]
2017	Phytochemistry and pharmacology	NS	The phytochemical part is briefly presented	[16]
2018	Pharmacology	NS	Only the antitumor activity was discussed	[17]
2020	Phytochemistry and pharmacology	NS		[18]
2021	Ethnomedicine	NS	Only the antitumor activity was discussed and the review is based mostly on *A. pilosa* preparation	[19]

**Table 2:** Flavonoids isolated from *Agrimonia pilosa* Ledeb.

No	Compounds	Molecules	Molecular weight	Plant part	References
1	Quercetin 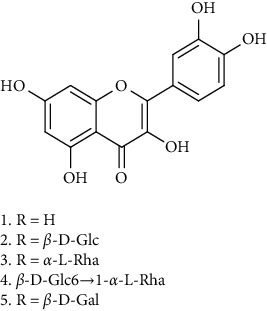	C_15_H_10_O_7_	302	Aerial parts	[15]
2	Isoquercitrin	C_21_H_20_O_12_	464	Aerial parts	[20]
3	Quercitrin	C_21_H_20_O_11_	448	Aerial parts	[20]
4	Rutin	C_27_H_30_O_16_	610	Aerial parts	[20]
5	Hyperin	C_21_H_20_O_12_	464	Aerial parts	[20]
6	Kaempferol 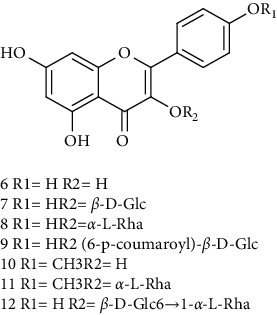	C_15_H_10_O_6_	386	Aerial parts	[22]
7	Kaempferol-3-O-*β*-d-glucopyranoside	C_21_H_20_O_11_	448	Aerial parts	[23]
8	Kaempferol-3-o-*α*-l-rhamnopyranoside	C_21_H_20_O_10_	432	Aerial parts	[23]
9	Tiliroside	C_30_H_26_O_13_	594	Aerial parts	[23]
10	Kaempferide	C_16_H_12_O_6_	300	Aerial parts	[24]
11	Kaempferide-3-O-*α*-L-rhamnopyranoside	C_22_H_22_O_10_	446	Aerial parts	[24]
12	Kaempferol-3-O-rutinoside	C_27_H_30_O_15_	594	Aerial parts	[24]
13	Apigenin 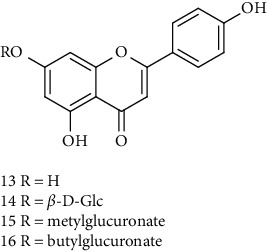	C_15_H_10_O_5_	270	Aerial parts	[25]
14	Apigenin-7-O-*β*-d-glucopyranoside	C_21_H_20_O_10_	432	Aerial parts	[15]
15	Apigenin-7-O-*β*-D-methylglucuronate	C_22_H_20_O_11_	460	Aerial parts	[25]
16	Apigenin-7-O-*β*-D-butylglucuronate	C_25_H_26_O_11_	502	Aerial parts	[25]
17	Luteolin-7-O-sophoroside 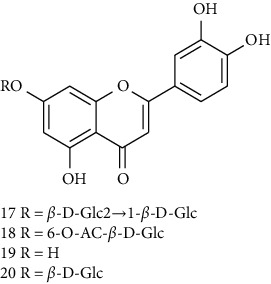	C_27_H_30_O_16_	610	Aerial parts	[15]
18	Luteolin-7-O-(6-O-acetyl)-D-glucopyranoside	C_23_H_22_O_11_	474	Aerial parts	[15]
19	Luteolin	C_15_H_10_O_6_	286	Aerial parts	[22]
20	Luteolin-7-O-*β*-D-glucopyranoside	C_21_H_20_O_11_	448	Aerial parts	[15]
21	Wogonin 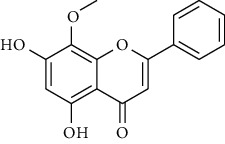	C_15_H_12_O_5_	272	Aerial parts	[25]
22	(+)-Catechin 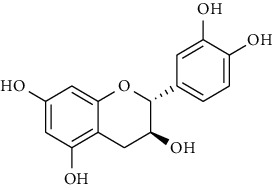	C_15_H_14_O_6_	290	Whole plant	[26]
23	Pilosanol A 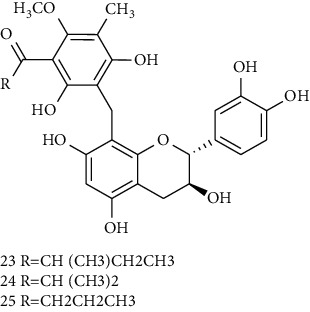	C_29_H_32_O_10_	540	Root	[27]
24	Pilosanol B	C_28_H_30_O_10_	526	Root	[27]
25	Pilosanol C	C_28_H_30_O_10_	526	Root	[27]
26	(2R, 3R)-(+)-Taxifolin 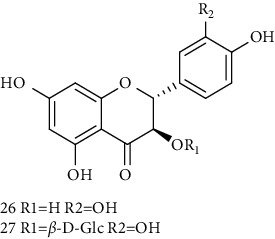	C_15_H_12_O_7_	304	Whole plant	[20]
27	(2R, 3R)-(+)-Taxifolin-3-O-*β*-D-glucopyranoside	C_21_H_22_O_12_	466	Aerial parts	[28]
28	(2S, 3S)-(−)-Taxifolin 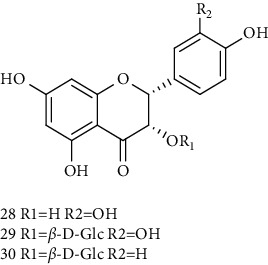	C_15_H_12_O_7_	304	Whole plant	[26]
29	(2S, 3S)–(−)-Taxifolin-3-O-*β*-D-glucopyranoside	C_21_H_22_O_12_	466	Aerial parts	[28]
30	(−)-Aromadendrin-3-O-*β*-D-glucopyranoside	C_21_H_22_O_11_	450	Aerial parts	[23]
31	Dehydrodicatechin A 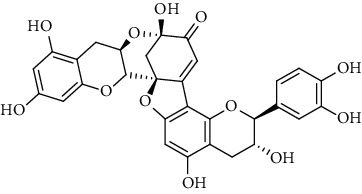	C_30_H_24_O_12_	576	Whole plant	[26]
32	Agriflavone 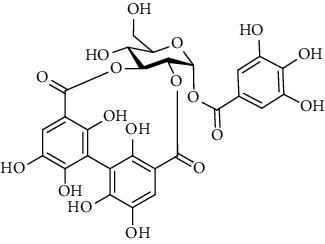	C_27_H_28_O_15_	593	Aerial parts	[29]
33	Vitexin 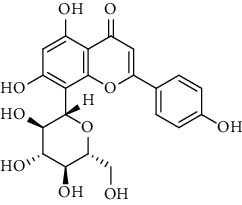	C_21_H_20_O_10_	432	Aerial parts	[30]
34	Isovitexin 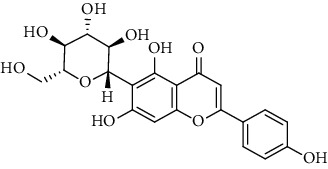	C_21_H_20_O_10_	432	Aerial parts	[30]
35	Dihydrodehydro-diconiferyl alcohol 9′-O-3-D-glucoside 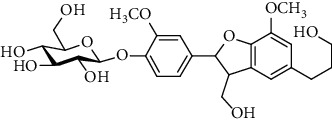	C_26_H_34_O_11_	522	Aerial parts	[29]
36	Dihydrokaempferol 3-O-*β*-D-glucoside 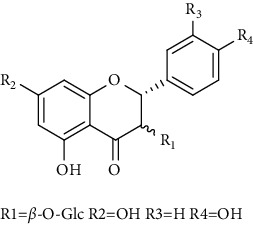	C_21_H_22_O_11_	450	Aerial parts	[31]
37	(2S, 3R)-dihydrokaempferol 3-O-*β*-D-glucoside 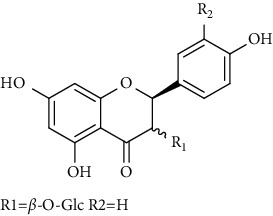	C_21_H_22_O_11_	450	Aerial parts	[31]

**Table 3:** Isocoumarins isolated from *Agrimonia pilosa* Ledeb.

No	Compounds	Molecules	Molecular weight	Plant part	References
1	Agrimonolide 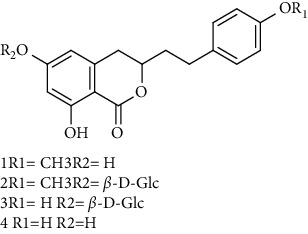	C_18_H_18_O_5_	314	Root	[32]
2	Agrimonolide-6-O-*β*-D-glucopyranoside	C_24_H_28_O_10_	476	Root	[23]
3	Desmethylagrimonolide-6-O-*β*-D-glucopyranoside	C_23_H_26_O_10_	462	Aerial parts	[23]
4	Desmethylagrimonolide	C_17_H_16_O_5_	300	Whole plant	[4]
5	(3S)-Agrimonolide-6-O-*α*-L-arabinofuranosyl-(1 ⟶ 6)-*β*-D-glucopyranoside 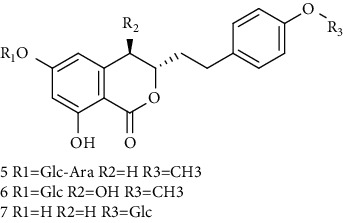	C_29_H_36_O_14_	609	Whole plant	[33]
6	(3S, 4R)-4-Hydroxyagrimonolide-6-O-*β*-D-glucopyranoside	C_24_H_28_O_11_	493	Whole plant	[33]
7	(3S)-Desmethylagrimonolide-4ʹ-O-*β*-D-glucopyranoside	C_23_H_26_O_10_	462	Whole plant	[33]
8	(3S)-Agrimonolide-6-(60ʹ-galloyl)-O-b-D-glucopyranoside 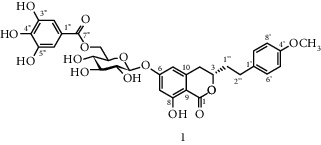	C_31_H_32_O_14_	629	Aerial parts	[34]

**Table 4:** Triterpenes isolated from *Agrimonia pilosa* Ledeb.

No	Compounds	Molecules	Molecular weight	Plant part	References
1	Corosolic acid 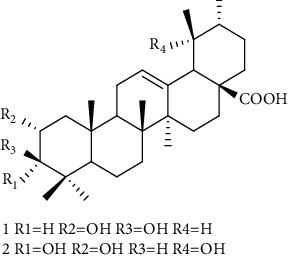	C_30_H_48_O_4_	472	Aerial parts	[38]
2	Euscaphic acid	C_30_H_48_O_5_	488	Aerial parts	[15]
3	Ursolic acid 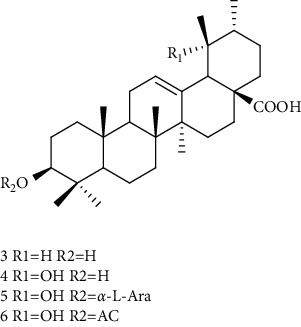	C_30_H_48_O_3_	456	Aerial parts	[38]
4	Pomolic acid	C_30_H_48_O_4_	472	Aerial parts	[38]
5	Ziyu-glucoside II	C_35_H_56_O_8_	604	Aerial parts	[39]
6	3-O-acetyl pomolic acid	C_32_H_50_O_5_	514	Aerial parts	[39]
7	Rosamultin 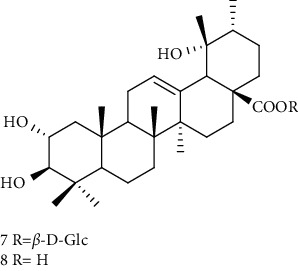	C_36_H_58_O_10_	650	Aerial parts	[38]
8	Tormentic acid	C_30_H_48_O_5_	488	Aerial parts	[38]
9	1*β*, 2*α*, 3*β*, 19*α*-Terahydroxyurs-12-en-28-oic acid 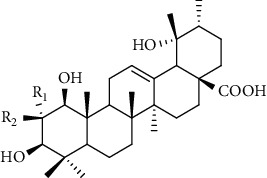	C_30_H_48_O_6_	504	Aerial parts	[39]
10	1*β*, 2*α*, 3*β*, 19*α*-Terahydroxyurs-12-en-28-oic acid	C_30_H_48_O_6_	504	Whole plant	[40]
11	27-Hydroxy-*α*-amyrin 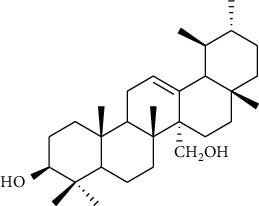	C_30_H_50_O_2_	442	Whole plant	[40]

**Table 5:** Phloroglucinol derivatives isolated from *Agrimonia pilosa* Ledeb.

No	Compounds	Molecules	Molecular weight	Plant part	References
1	Agrimol A 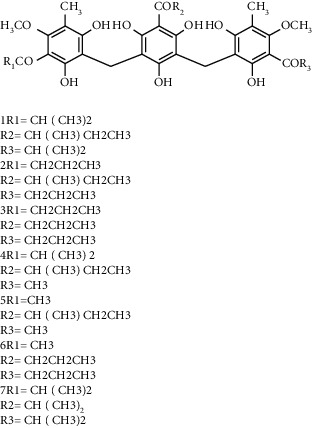	C_37_H_46_O_12_	682	Whole plant	[43]
2	Agrimol B	C_37_H_46_O_12_	682	Whole plant	[43]
3	Agrimol C	C_36_H_44_O_12_	668	Whole plant	[43]
4	Agrimol D	C_35_H_42_O_12_	654	Whole plant	[43]
5	Agrimol E	C_33_H_38_O_12_	626	Whole plant	[43]
6	Agrimol F	C_34_H_40_O_12_	640	Whole plant	[46]
7	Agrimol G	C_36_H_44_O_12_	668	Whole plant	[43]
8	Pilosanol N 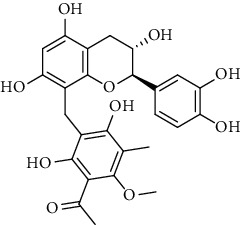	C_26_H_26_O_10_	498	Foliage	[47]
9	Agrimophol 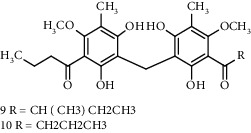	C_25_H_32_O_8_	460	Root	[44]
10	Pseudoaspidin	C_24_H_30_O_8_	446	Root	[44]
11	Agripinol A 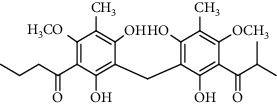	C_25_H_31_O_8_	459	Aerial parts	[45]
12	Agripinol B 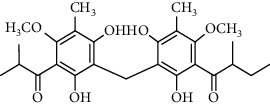	C_26_H_33_O_8_	473	Aerial parts	[45]
13	Agripinol C 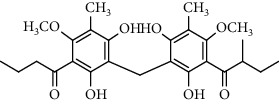	C_26_H_33_O_8_	473	Aerial parts	[45]
14	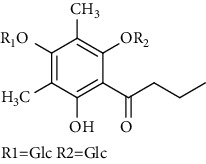	C_25_H_38_O_14_	563	Aerial parts	[48]
15	3,5-Dimethyl-a-methylbutyrylphloroglucinol-2,4-O-b-D-diglucopyranoside 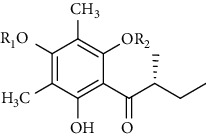	C_25_H_38_O_14_	563	Aerial parts	[48]
16	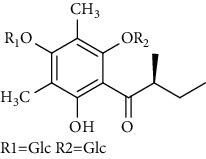	C_25_H_38_O_14_	563	Aerial parts	[48]
17	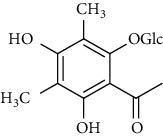	C_16_H_24_O_10_	376	Aerial parts	[48]
18	Agrimone A 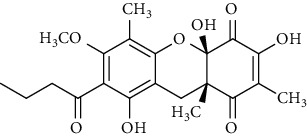	C_21_H_25_O_8_	405	Whole plant	[49]
19	Agrimone B 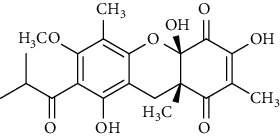	C_21_H_25_O_8_	405	Whole plant	[49]
20	Agrimone C 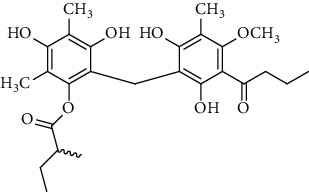	C_26_H_34_O_8_	475	Whole plant	[49]
21	Agrimone D 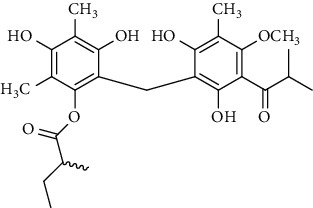	C_26_H_34_O_8_	475	Whole plant	[49]
22	Agrimone E 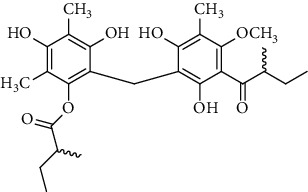	C_27_H_36_O_8_	489	Whole plant	[49]

**Table 6:** Tannins and organic acids isolated from *Agrimonia pilosa* Ledeb.

No	Compounds	Molecules	Molecular weight	Plant part	References
1	Potentillin 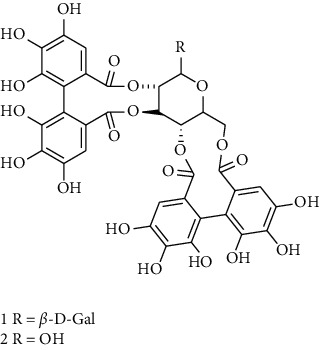	C_41_H_28_O_25_	920	Root	[50]
2	Pedunculagin	C_34_H_24_O_22_	784	Root	[50]
3	Casuarinin 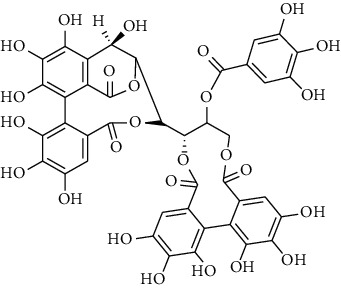	C_41_H_28_O_26_	936	Root	[50]
4	Alagrimonic A 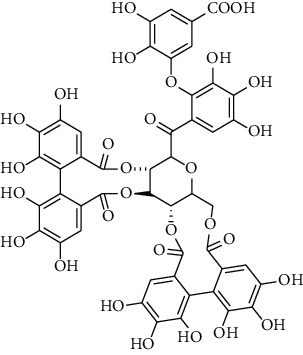	C_47_H_39_O_31_	1099	Root	[50]
5	Alagrimonic B 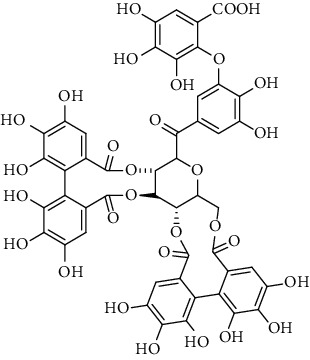	C_47_H_39_O_32_	1115	Root	[50]
6	Agrimoniin 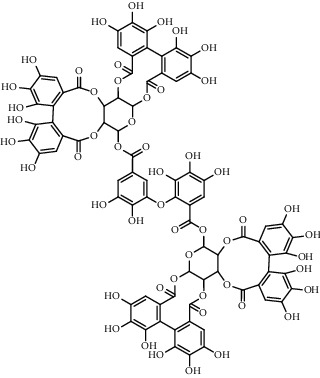	C_82_H_54_O_52_	1871	Root	[50]
7	Gallic acid 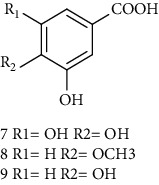	C_7_H_6_O_5_	170	Aerial parts	[20]
8	Isovanillic acid	C_8_H_8_O_4_	168	Aerial parts	[26]
9	Protocatechuic acid	C_7_H_6_O_4_	154	Aerial parts	[26]
10	Protocatechuic aldehyde 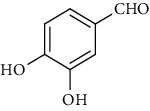	C_7_H_6_O_3_	138	Aerial parts	[26]
11	Agritannin 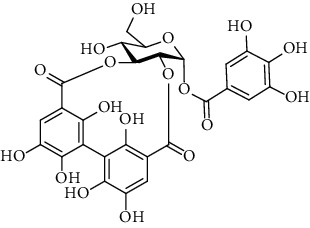	C_27_H_22_O_18_	657	Aerial parts	[29]
12	Ellagic acid 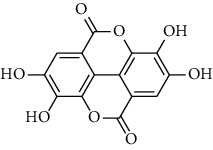	C_14_H_6_O_8_	302	Aerial parts	[29]

**Table 7:** Antitumor activities of components from *Agrimonia pilosa* Ledeb.

*A. pilosa* extract	Experimental model	Test dose range	Contrast	Route of administration	Pharmacological action	Mechanism of action	References
Quercetin	AGS cells	6.25, 12.5, 25, 50, and 100 *μ*M	SN-38 (6.25, 12.5, 25, 50, and 100 *μ*M)	NS	Sensitize AGS to SN-38	NS	[55]
Quercetin	AGS inoculate BALB/c nude mice	20 mg/kg, three times a week + IRI; 10 mg/kg, once a week	IRI; 10 mg/kg, once a week 20 mg/kg, three times a week	i.p.	Sensitize AGS to SN-38	NS	[55]
Agrimonolide	AGS cells	5, 10, 20, and 40 *μ*M	Negative control	NS	Apoptosis-inducing	Bcl-2/Bax↑, p-p38↑ and caspase-3 protease activation	[56]
Beta-carotene	AGS cells	0, 20, 50, and 100 *μ*mol/L	Negative control	NS	Apoptosis-inducing and DNA fragmentation	Bcl-2/Bax↑,p-53↑	[1]
Agrimol B	PC-3 cells	0, 6.25, 12.5, and 25 *μ*mol/L	Negative control	NS	Arrest cancer cells at G0 phase	p27↑, SKP2↓，cMYC↓	[57]
Agrimol B	A549 cells	0, 6.25, 12.5, and 25 *μ*mol/L	Negative control	NS	Arrest cancer cells at G0 phase	SKP2↓, cMYC↓, SPT16↓ and SSRP1↓, p27↑	[57]
Ellagic acid	PC-3 cells	0, 6.25, 12.5, and 25 *μ*mol/L	Negative control	NS	Arrest cancer cells at G0 phase	c-MYC↓, SKP2↓, SPT16↓, SSRP1↓, p27↑	[57]
Ellagic acid	A549 cells	0, 6.25, 12.5, and 25 *μ*mol/L	Negative control	NS	Arrest cancer cells at G0 phase	c-MYC↓, SKP2↓, SPT16↓, SSRP1↓, CRM1↓, p27↑	[57]
Agrimol B	PC-3 inoculated male BALB/c nude mice	10 mg/kg	Negative control	p.o.	Tumor growth reducing	NS	[57]
Apigenin	PC-3 cells	20, 40 *μ*M	Negative control	NS	Apoptosis inducing	HDACs↓, especially HDAC1↓ and HDAC3↓	[58]
Apigenin	PC-3 xenografts in athymic nude mice	20 and 50 *μ*g/mouse/day	Negative control	p.o.	Tumor growth reducing	HDACs↓ p21/waf1↑, Bax/bcl2↑	[58]
Quercetin	MCF-7 cells	0–100 *μ*g/ml, IC50 = 0.87 *μ*g/mL	Negative control	NS	Cytotoxicity	May be related to the presence of 2,3-double bond in ring C, carbonyl group at C-4 and ortho-hydroxylation in ring B	[59]
Agrimophol	K562 cells	0.1, 1, 10 mg/ml	Vincristine (50 IU/ml)	NS	Apoptosis inducing	NS	[60]
Agripinol A	HCT-116, MDA-MB-231 and PC-3 cells	(IC50 = 12.34 ± 0.93, 5.44 ± 0.35, 9.47 ± 0.70, 14.29 ± 1.24 *μ*g/ml, respectively)	Fluorouracil	NS	Cytotoxicity	NS	[45]
Agripinol B	HCT-116, MDA-MB-231 and PC-3 cells	(IC50 = 12.34 ± 0.93, 5.44 ± 0.35, 9.47 ± 0.70, 14.29 ± 1.24 *μ*g/ml, respectively)	Fluorouracil	NS	Cytotoxicity	NS	[45]
Agripinol C	HCT-116, MDA-MB-231 and PC-3 cells	(IC50 = 12.63 ± 1.40, 2.12 ± 0.16, 7.50 ± 0.86, 9.85 ± 1.08 *μ*g/ml, respectively)	Fluorouracil	NS	Cytotoxicity	NS	[45]
Agrimoniin	MM2 inoculated C3H/H e and BALB/ c mice	1, 3, 10, 30 mg/kg	Negative control	i.p.	Prolonged the life span of mice bearing MM2	Direct inhibit tumor cell activity and increased the number of peripheral white blood cells and the ratio of monocytes	[61]
Quercetin and hyperoside in combination (1 : 1 ratio)	786-O renal cancer cells	3.8–60 *μ*g/ml	Negative control	NS	Cancer cell proliferation inhibition	ZBTB10↑ Sp1, Sp3, and Sp4 mRNA↓	[62]

**Table 8:** Free radical-scavenging and antioxidant activities of components from *A. pilosa*.

*A. pilosa* extract	Experimental model	Test dose range	Contrast	Route of administration	Pharmacological action	Mechanism of action	References
*A. pilosa* flavonoids (36.45 mg/ml)	FRAP working fluid	FRAP = 56.87mg^−1^	Vit C (FRAP = 45.47 mg^−1^)	NS	Antioxidant activities	NS	[63]
*A. pilosa* flavonoids (316.53 ± 6.37 mg/g)	100 *μ*L sample in methanol was mixed with 1.9 mL of 0.1 mM DPPH in ethanol	0.25, 0.5, 2.5, 5.0, 25.0, 50.0, 100.0 *μ*g/mL	2,6-Di-tert-butyl-4-methylphenol	NS	DPPH scavenging activity	NS	[64]
*A. pilosa* flavonoids 316.53 ± 6.37 mg/g	0.75 mM 1,10-phenanthroline and 0.75 mM FeSO4 were prepared in 0.05 M phosphate buffer (pH 7.4) and mixed thoroughly (method described by De Avellar and jin)	5.0, 10.0, 50.0, 100.0, 500.0, 1000.0, 2000.0 *μ*g/mL	Negative control	NS	Hydroxyl radical scavenging activity	NS	[64]
*A. pilosa* aqueous extract	Low immunity mice	100, 300, 1000 mg/kg	Negative control	p.o.	Antioxidant	MDA↑、SOD↑	[15]
Protocatechuic acid	The method of Brand-Williams et al.	15 *μ*M	Negative control	NS	DPPH free radical scavenging	Providing hydrogen atoms or electron donation	[65]
Protocatechuic acid	Generated by the deoxyribose method (Halliwell 1987)	15 *μ*M	Negative control	NS	Superoxide radical (O2-) scavenging	NS	[65]
*A. pilosa* flavonoids 316.53 ± 6.37 mg/g	Supercoiled plasmid pBR322 DNA	0.1 mM, 1.0 mM	Negative control	NS	Against DNA oxidative damage	NS	[3]
Protocatechuic acid	Male albino rats of Wistar strain	10, 20 mg/kg	Negative control	p.o.	Protects damaged rat liver cells	Enhancing antioxidant capacity and enhancing stage II enzyme activity through the Nrf-2 pathway	[66]

**Table 9:** Anti-inflammatory activity of components from *A. pilosa*.

A. pilosa extract	Experimental model	Test dose range	Contrast	Route of administration	Pharmacological action	Mechanism of action	References
Agrimonolide	LPS-induced RAW 264.7 cells	0, 20, 40, 60, 80 *μ*g/ml	Negative control	NS	Anti-inflammatory	NO scavenging, COX-2/inos↓, NF-*κ*B ↓, MAPKs↓, JAK-STATs↓	[35]
Agrimonolide-6-O-*β*-D-glucopyranoside	LPS-induced RAW 264.7 cells	50, 100, 200 *μ*g/ml	4-Ethyl-2-hydroxyamino-5-nitro-3-hexenamide (200 *μ*M)	NS	NO scavenging	NS	[47]
Agrimonolide-6-O-*β*-D-glucopyranoside	LPS-induced RAW 264.7 cells	25, 50, 100 *μ*g/ml	Negative control	NS	NO production decreasing	NS	[35]
Pilosanol N	LPS/IFN-*γ*-induced RAW264.7 macrophages	25, 50, 100 *μ*g/ml	NOR3 (200 *μ*M)	NS	NO scavenging	May contribute to the catechol group (3′, 4′-OH) of the B ring in the structure	[67]
Pilosanol N	LPS/IFN-*γ*-induced RAW 264.7 macrophages	25, 50, 100 *μ*g/ml	IFN-*γ* and l-arginine	NS	NO production decreasing	iNOS↑and may also contribute to NF-*κ*B/NO signaling disrupting	[67]

**Table 10:** Antidiabetic activity and lipid metabolism regulation effects of components from *A. pilosa*.

A. pilosa extract	Experimental model	Test dose range	Contrast	Route of administration	Pharmacological action	Mechanism of action	References
Apigenin 7-O-*β*-D-glucuronide	NS	IC50 = 7.14 ± 1.75 *μ*M	Ursolic acid (IC50 = 9.43 ± 0.14 *μ*M)	NS	Improve insulin resistance	PTP1B inhibition	[29]
Ellagic acid	NS	IC50 = 7.73 ± 0.24 *μ*M	Ursolic acid (IC50 = 9.43 ± 0.14 *μ*M)	NS	Improve insulin resistance	PTP1B inhibition	[29]
Quercetin	NS	IC50 = 28.7 ± 1.2 *μ*M	Acarbose (IC50 = 45.2 ± 1.2 *μ*M)	NS	Glycogen decomposition and glucose regulation	Competitively *α*-glucosidase inhibition	[30]
Agrimonolide	NS	IC50 = 24.2 *μ*M	Acarbose (IC50 = 45.2 ± 1.2 *μ*M)	NS	Glycogen decomposition and glucose regulation	Noncompetitively *α*-glucosidase inhibition	[30]
Desmethylagrimonolide	NS	IC50 = 37.4 *μ*M	Acarbose (IC50 = 45.2 ± 1.2 *μ*M)	NS	Improve insulin resistance of HepG2 cells	Non-competitively *α*-glucosidase inhibition	[30]
Agrimonolide	Insulin-resistance HepG2 cells	IC50 = 8.3 ± 0.6 *μ*M	Metformin (IC50 = 18.6 ± 0.8 *μ*M)	NS	Improve insulin resistance of HepG2 cells	Phosphoenolpyruvate carboxykinase inhibition	[68]
Agrimonolide	Insulin-resistance HepG2 cells	IC50 = 11.6 ± 0.8 *μ*M	Metformin (IC50 = 12.4 ± 1.6 *μ*M)	NS	Improve insulin resistance of HepG2 cells	Hepatic glucose-6-phosphatase inhibition	[68]
Total triterpenoids of *Agrimonia pilosa* Ledeb (415.97 ± 5.15 mg/g)	3T3-L1 cells	1, 5, 25, and 125 *μ*g/ml	Pioglitazone (10 *μ*M)	NS	Insulin sensitization effects with low lipid formation effects	PPAR*γ*↑SREBP-1↑C/EBP*α*↑, thus upregulating adiponectin and GLUT4 mRNA expression	[69]
Total triterpenoids of *Agrimonia pilosa* Ledeb (the content is not clear)	High sugar concentration-induced IR-HepG2 cells	5, 25, 50, 15, 100, 125 *μ*g/ml	Rosig (30 *μ*M)	NS	Regulation of lipid metabolism	Improving oxidative stress and regulating the JNK and IRS pathways, thus improved glucose metabolism in IR-HepG2 cells	[41]
Agrimol B	3T3-L1 cells	3, 10 *μ*g/ml IC50 = 3.35 ± 0.32 *μ*M	Resveratrol (50 *μ*M) and berberine (10 *μ*M)	NS	Regulation of lipid metabolism	PPAR↓、C/EBP*α*↓、FAS↓、UCP-1 and apoE↓, thus inhibited 3T3-L1 adipocyte differentiation	[70]

**Table 11:** Anthelmintic activity of components from *A. pilosa*.

A. pilosa extract	Experimental model	Test dose range	Contrast	Route of administration	Pharmacological action	Mechanism of action	References
Agrimophol	Cysticercus cellulosae with intact cyst in vitro	2 × 10^−4^, 5 × 10^−4^, 2 × 10^−5^, 2 × 10^−6^, 2 × 10^−7^ *μ*g/ml	Negative control	NS	Antitapeworm	Aerobic and anaerobic metabolism inhibition through direct contact with insects	[71]

Agrimol G	Adult *H. contortus* parasites	150, 300, 600, and 1200 *μ*g/ml	Albendazole (380 *μ*g/ml) Ivermectin (500 *μ*g/ml)	Coincubation	Antitapeworm	Microtubule aggregation inhibition	[72]

**Table 12:** Hepatocyte protection activity of components from *A. pilosa*.

*A. pilosa* extract	Experimental model	Test dose range	Contrast	Route of administration	Pharmacological action	Mechanism of action	References
Agrimonolide	Tacrine-induced human liver-derived hep G2 cells	1–100 *μ*M EC_50_ = 88.2 ± 2.8 *μ*M	Silybin	NS	Hepatocyte protection effects	NS	[5]

Agrimonolide	Tert-butyl hydroperoxide-induced rat primary hepatocytes	1–100 *μ*M EC50 = 37.7 ± 1.6 *μ*M	Silybin	NS	Hepatocyte protection effects	NS	[5]

Agrimonolide	H_2_O_2_ induced HepG2 cells	50, 100, 200 *μ*M	Negative control	NS	Oxidative stress reducing and hepatocyte protection effects	Inducing heme oxygenase-1 and Nrf2 expression and inhibiting Kelch-like ECH-associated protein 1 expression	[73]

Desmethylagrimonolide	H_2_O_2_ induced HepG2 cells	50, 100, 200 *μ*M	Negative control	NS	Oxidative stress reducing and hepatocyte protection effects	Inducing heme oxygenase-1 and Nrf2 expression and inhibiting Kelch-like ECH-associated protein 1 expression	[73]

**Table 13:** Antimicrobial activity of components from *A. pilosa*.

A. pilosa extract	Experimental model	Test dose range	Contrast	Route of administration	Pharmacological action	Mechanism of action	References
Agrimol C	MIC against *Staphylococcus aureus* 2o9P, *Bacillus cereus* var. mycoides, and Nocardia gardneri. Method described by takagi et al.	MIC = 50, 25, 100 *μ*M, respectively	Negative control	NS	Antimicrobial activity	NS	[46]

Agrimol F	MIC against *Staphylococcus aureus* 2o9P, *Bacillus cereus* var. mycoides, and *Nocardia gardneri*. Method described by Takagi et al.	MIC = 25, 25, 100 *μ*M, respectively	Negative control	NS	Antimicrobial activity	NS	[46]

Agrimol G	MIC against *Staphylococcus aureus* 2o9P, *Bacillus cereus* var. mycoides, and *Nocardia gardneri*. Method described by Takagi et al.	MIC = 12.5, 50, 100 *μ*M, respectively	Negative control	NS	Antimicrobial activity	NS	[46]

Agrimophol	MIC against *Staphylococcus aureus* 2o9P, *Bacillus cereus* var. mycoides, and *Nocardia gardneri*. Method described by Takagi et al.	MIC = 3.13,6.25,100 *μ*M, respectively	Negative control	NS	Antimicrobial activity	NS	[46]

## Abbreviations

TCM: Traditional Chinese medicine
HDAC: Histone deacetylase
Bcl-2: B-cell lymphoma-2
T2DM: Type 2 diabetes mellitus
PTP: Protein tyrosine phosphatase
IL: Interleukin
TNF-*α*: Tumor necrosis factor-*α*
AchE: Acetylcholinesterase
Nrf2: Nuclear factor-E2-related factor 2
MAPK: Mitogen-activated protein kinase
NO: Nitric oxide
iNOS: Inducible nitric oxide synthase
COX-2: Cyclooxygenase-2
LPS: Lipopolysaccharide
JNK: C-Jun *n*-terminal kinase
STAT: Signal transducer and activator of transcription
IR: Insulin resistance
Bax: Bcl-2-associated X
IRS: Insulin receptor substrate
PPAR*γ*: Peroxisome proliferator-activated receptor *γ*
SIRT1: Silent mating-type information regulation 2 homolog 1
c-MYC: Cellular-myelocytomatosis viral oncogene
DPPH: 2,2-Diphenyl-1-picrylhydrazyl
MDA: Malondialdehyde
ERs: Estrogen receptors.
